# PIGT promotes cell growth, glycolysis, and metastasis in bladder cancer by modulating GLUT1 glycosylation and membrane trafficking

**DOI:** 10.1186/s12967-023-04805-0

**Published:** 2024-01-02

**Authors:** Mingyue Tan, Qi Pan, Chao Yu, Xinyu Zhai, Jianyi Gu, Le Tao, Dongliang Xu

**Affiliations:** 1 Urology Center, Shuguang Hospital, Shanghai University of Traditional Chinese Medicine, No. 528, Zhangheng Road, Pudong New Area, Shanghai, 201203 China; 2 Department of Urology, Shanghai General Hospital, Shanghai Jiao Tong University School of Medicine, Shanghai, 200080 China; 3 Department of Urology and Andrology, Longhua Hospital Affiliated to Shanghai University of Traditional Chinese Medicine, Shanghai, 200032 China; 4 Department of Urology, Renji Hospital, Shanghai Jiao Tong University School of Medicine, No.160 Pujian Road, Pudong New Area, Shanghai, 200127 China

**Keywords:** Bladder cancer, RNA methylation, phosphatidylinositol Glycan Anchor Biosynthesis Class T, Glucose transporter 1, Glycolysis

## Abstract

**Background:**

Bladder cancer is very common worldwide. PIGT is a subunit of the glycosylphosphatidylinositol transamidase which involves in tumorigenesis and invasiveness. m6A modification of mRNA has been linked to cell proliferation, tumor progression and other biological events. However, how PIGT is regulated and what is the function of PIGT in bladder cancer remains to be elucidated.

**Methods:**

PIGT was silenced or overexpressed to study its role in regulating bladder cancer. Cell proliferation and invasion were examined with the Cell Counting Kit-8, colony formation and Transwell assay, respectively. Cellular oxygen consumption rates or extracellular acidification rates were detected by a XF24 Analyzer. Quantitative RT-PCR and immunoblots were performed to detect mRNA and protein levels.

**Results:**

PIGT was overexpressed in bladder cancer. Silencing PIGT inhibited cell proliferation, oxidative phosphorylation, and glycolysis. Overexpressing PIGT promoted cell proliferation, oxidative phosphorylation, glycolysis in vitro and tumor metastasis in vivo by activating glucose transporter 1 (GLUT1). PIGT also promoted GLUT1 glycosylation and membrane trafficking. Wilms’ tumor 1-associated protein (WTAP) mediated PIGT m6A modification, and m6A reader, insulin-like growth factor 2 mRNA-binding protein (IGF2BP2), binds to the methylated PIGT to promote the stability of PIGT, leading to up-regulation of PIGT.

**Conclusion:**

WTAP mediates PIGT m6A modification to increase the stability of PIGT via the IGF2BP2, which enhances cell proliferation, glycolysis, and metastasis in bladder cancer by modulating GLUT1 glycosylation and membrane trafficking.

**Supplementary Information:**

The online version contains supplementary material available at 10.1186/s12967-023-04805-0.

## Background

Bladder cancer is the tenth most commoncause of cancer globally and the thirteenth most common cause of mortality from cancer [1]. In 2020, 573,278 people were newly diagnosed with and 212,536 patients died of bladder cancer worldwide [2] and the incidence of bladder cancer is steadily rising across the globe representing 3% of all cancers in 2020 [2]. Possible risk factors include smoking, occupational exposures, sex, age, parasitic infection, chronic inflammation, and genetic factors [3]. Chemotherapy and radical cystectomy are used for patients of invasive bladder cancer [4, 5]. Although advances have been made in treating bladder cancer, improved understanding of bladder cancer will definitely evolve the way it is diagnosed and treated.

PIGT (Phosphatidylinositol Glycan Anchor Biosynthesis Class T) is a subunit of glycosylphosphatidylinositol (GPI) transamidase (GPIT), which facilitates the modification of proteins by GPI [6]. PIGT mutation can cause seizures, facial dysmorphism, and hypotonia [7]. Overexpression of PIGT has been shown to induce tumorigenesis and contribute to invasion in breast cancer [8, 9]. Guo et al. have demonstrated that another subunit of the GPI transamidase complex, PIGU, was downregulated in differentiated thyroid carcinoma [10] and overexpressed in hepatocellular carcinoma, and might be involved in cell cycle-related biological processes in hepatocellular carcinoma [11]. *N*6-methyladenosine (m6A) is a chemical derivative of adenosine in RNA that plays important, wide-ranging roles in gene expression [12]. m6A occurs in most eukaryotes. The effects of m6A are mediated by m6A readers, writers, and erasers [13]. Studies have linked m6A to cell proliferation, cancer progression etc. Wang et al. indicated that m6A promotes breast cancer progression by targeting Bcl-2 [14]. In another study, Shriwas et al. indicated that DDX3 regulates cisplatin resistance via ALKBH5-mediated m(6)A-demethylation of FOXM1 and NANOG [15]. Chen et al. have demonstrated that regulation of m6A RNA methylation affects malignant progression of bladder cancer, and suggested that m6A RNA methylation regulators may be promising prognostic biomarkers [16]. m6A RNA methylation also enhances bladder cancer progression [17]. However, the roles of PIGT and m6A RNA methylation in bladder cancer are poorly understood and remain to be elucidated.

Glycolysis provides energy for cells [18]. Found in almost every tissue, glucose transporter 1 (GLUT1) controls glucose uptake and glycolysis to regulate tumorigenesis and tumor progression [19]. For instance, Xiao et al. demonstrated that GLUT1 regulates glycolysis and cell growth in prostate cancer [19]. GLUT1 also plays a very important role in bladder cancer. Al-Maghrabi et al. demonstrated that increased GLUT1 expression has been found in urinary bladder cancer [20]. Wang et al. indicated that uniquitination and degradation of GLUT1 could restrict tumor progression in bladder cancer [21]. Studies also showed that Sirtuin 1 increases GLUT1 level and bladder cancer progression via regulating glucose uptake [22]. However, the specific function and clinical significance of GLUT1 in bladder cancer are largely unknown. Therefore, we aim to study the role of PIGT and GLUT1 in bladder cancer and its underlying mechanisms.

## Materials and methods

### Ethical approval

The study was conducted under the approval of the Ethics Committee of the Shuguang Hospital, and all procedures in the animal experiment were conducted in accordance with the *Guide for the Care and Use of Laboratory Animals*.

### Bioinformatics analysis

Single-cell RNA-seq data was obtained from Bioproject (www.ncbi.nlm.nih.gov/bioproject/?term=PRJNA66201). Unique molecular identifier (UMI) count matrix was converted to anndata objects with Scanpy package v1.4.4. Batch was corrected by utilization of the RunHarmony function in its R package (version 1.0). Uniform Manifold Approximation and Projection (UMAP) was run to visualize data. Effect size was evaluated using Cohen’s d statistic to estimate the magnitude of differentially expressed genes. RNAseq data related to PIGT and the prognostic value of PIGT were examined in patients with bladder cancer from The Cancer Genome Atlas (TCGA) dataset via the Gene Expression Profiling Interactive Analysis (GEPIA), including 404 tumor and 19 adjacent normal samples. Gene set enrichment analysis (GSEA) algorithm was used to find the enriched pathways between PIGT-high and PIGT-low groups using the median transcript per million (TPM) as the cutoff point.

### Clinical samples

Resected tumor and corresponding adjacent-normal tissues were obtained from 25 patients with bladder cancer who underwent surgery at the Shuguang Hospital. These experiments were approved by Ethics Committee of the Shuguang Hospital and informed consents were received. The human bladder cancer tissue microarrays (Shanghai Outdo Biotech Co., Ltd., China) including 111 tumor and 15 adjacent normal samples were used to detect PIGT, WTAP, and GLUT1 protein expression through immunohistochemistry (IHC). Clinicopathological features of bladder cancer patients are shown in Additional file 1: Table S1.

### IHC

The sectioned tissue specimens (5-µm thick) from tissue microarrays were blocked with 0.3% hydrogen peroxide for 15 min at 25 °C and incubated with anti-PIGT (1:1000; ab237800; Abcam, Waltham, MA, USA), anti-GLUT1 (1:500; ab115730; Abcam), or anti-WTAP antibody (1:100; ab195380; Abcam), followed by horseradish peroxidase (HRP)-conjugated anti-IgG antibody (D-3004; Long Island Biotech, Shanghai, China). Immunoreactivity was scored using the H-score system by two investigators based on the percentage of positively stained cells (graded on a scale of 0–4: 0, < 5%; 1, 5%-25%; 2, 25%-50%; 3, 50%-75%; 4, > 75%) and the intensity of staining (graded on a scale of 0–3: 0, negative; 1, weak; 2, moderate; 3, strong), which ranged from 0–12 [23]. Based on immunostaining, the patients were divided into low expression (H-score < 4) or high expression (H-score ≥ 4) group.

### Bladder cancer cell lines and culture conditions

253 J (XY-XB-2404; Shanghai Xuanya Biotechnology Co., Ltd, China), 5637 (HTB-9; ATCC, Manassas, VA, USA), BIU-87 (ZY-1004H; Shanghai Zeye Biotechnology Co., Ltd, China), T24 (HTB-4; ATCC) and SCABER (HTB-3; ATCC) cells were cultured in DMEM (11,960,044) or RPMI-1640 (11,875,119) with 10% fetal bovine serum (10,099,158; all from Thermo Fisher Scientific, Rockford, IL, USA) and incubated at 37 °C. They were authenticated on July 11, 2021 using short tandem repeat profiling and all experiments were performed with mycoplasma-free cells.

### Lentivirus construction and siRNA transfection

The control pLVX-Puro lentivirus, PIGT overexpression lentivirus and WTAP overexpression lentivirus were obtained from Generay Biotech (Shanghai) Co., Ltd, China. The control pLKO.1 shRNA lentivirus, PIGT shRNA lentivirus and GLUT1 shRNA lentivirus were obtained from Obio Technology Company (Shanghai, China). Small interfering RNAs (Shanghai GenePharma Co., Ltd, China) were transfected using lipo2000 (11,668,500; Invitrogen, Thermo Fisher Scientific). Empty vector and scramble siRNA were used as negative control. All the sequences of shRNA and siRNAs were listed in Additional file 1: Table S2.

### Cell growth

Cell viability was measured using Cell Counting Kit-8 (CCK-8) (CK04; Dojindo Molecular Technologies, Kumamoto, Japan). Briefly, cells were grown to the logarithmic phase, harvested, and seeded into 96-well plates at a cell density of 3 × 10^3^ cells per well. At 12, 24, and 48 h after treatment, 10 μL CCK-8 solution was added to each well and maintained the reaction time for 1 h. Absorbance was measured at 450 nm using a microplate reader and used for the calculation of cell viability.

### Colony formation assay

Forty-eight hours after treatment, cells (1 × 10^3^ cells per well) were seeded in 6 cm dishes and cultured for ten days. At the end of the incubation, colonies were fixed with formaldehyde for 15 min and stained with 0.5% crystal violet for 30 min. Colonies with 50 cells or more were counted.

### Extracellular flux (XF) analysis

With a Seahorse XF24 Analyzer, oxygen consumption rate (OCR) and extracellular acidification rate (ECAR) were recorded as previously described [24]. Briely, cells digested to a density of 1 × 10^4^/well, were seeded in XF24 culture plates (Agilent Technologies, Santa Clara, CA, USA), and were then placed in an incubator of 37 °C and 5% CO_2_ for 24 h. Around 1 h before detection, cells were shifted into an incubator without CO_2_, and culture medium was replaced by XF Base Medium (Agilent Technologies). OCR was measured using Seahorse XF Cell Mito Stress Test Kit (103,015–100; Agilent Technologies) and ECAR was measured using Seahorse XF Glycolytic Rate Assay Kit (103,344–100; Agilent Technologies).

### Measurement of lactate and ATP

The cells were seeded in 96-well plates at 3.5 × 10^3^ cells per well. After overnight incubation at 37℃, 5% CO_2_, the complete medium was changed to fresh medium (50 μl/well). After 24 h, the supernatant of cells was collected by centrifugation. Then, according to the manufacturer's instructions, the lactate release was determined using Lactic Acid assay kit (A019-2; Nanjing Jiancheng Bioengineering Institute, China). ATP content was measured with the ATP assay kit (A095; Nanjing Jiancheng Bioengineering Institute), as per the manufacturer's protocol. In brief, cells were seeded in the 6-well plate for 12–24 h. Then cells were harvested by using 200–300 μl lysis buffer and vortexed for 1 min. The supernatant was mixed with detection solution and then analysis for ATP concentration was normalized to the corresponding total protein amounts from each sample.

### Transwell assay

Cell migration and invasion abilities were assessed using Transwell assays. Cells were suspended in serum-free medium, and 5 × 10^4^ cells were seeded into the upper chambers of Transwell inserts (CLS3464-48EA; Corning Costar, Cambridge, MA, USA). A culture medium containing 10% fetal bovine serum was added to the lower chamber. Following an incubation period of 24–48 h, the Transwell chambers were immobilized using formaldehyde, followed by staining with 5% crystal violet. After rinsing with phosphate-buffered saline, the cells that had successfully migrated were captured and quantified. For the cell invasion assay, the same procedure was conducted, but the Transwell inserts were pre-coated with a Matrigel mixture before cell seeding.

### Quantitative RT-PCR (RT-qPCR)

RNA was isolated using TRIzol reagent (15,596,026; Thermo Fisher Scientific). The first-strand cDNA was synthesized using the PrimeScript RT Reagent Kit (RR047A; Takara Biomedical Technology (Beijing) Co., Ltd, China) as per manufacturer’s instructions. RT-qPCR was performed using SYBR Green PCR Master Mix (4,309,155; Thermo Fisher Scientific). The relative expression level of target genes was normalized to that of β-actin using the 2^−ΔΔCt^ method. Primer sequences were shown in Additional file 1: Table S3.

### Western blot

Proteins were extracted using radioimmunoprecipitation assay buffer (ab288006; Abcam) and quantified by BCA Protein Assay Kit (PICPI23223; Thermo Fisher Scientific). 40 μg of protein was separated by sodium dodecyl-sulfate–polyacrylamide gel electrophoresis and then transferred to polyvinylidene fluoride membranes. After blocking, membranes were incubated with antibodies against PIGT (1:500; 16,906–1-AP; Proteintech Group, Inc., Rosemont, IL, USA), GLUT1 (1:30,000; ab115730; Abcam), METTL3 (1:1000; ab195352; Abcam), METTL14 (1:500; ab220030; Abcam), WTAP (1:1000; ab195380; Abcam), IGF2BP1 (1:1000; ab184305; Abcam), IGF2BP2 (1:1000; ab129071; Abcam), IGF2BP3 (1:1000; ab177477; Abcam), and β-actin (1:1000; ab8226; Abcam) at room temperature for 1 h. Secondary antibodies were labeled with HRP (1:1000; A0208, A0216; Beyotime Biotechnology, Shanghai, China). Specific signals were visualized using an enhanced chemiluminescence substrate kit (P0018F, Beyotime Biotechnology).

### Immunofluorescence microscopy

After being fixed and permeabilized, cells were fixed, permeabilized, blocked, and incubated with anti-GLUT1 (1:500; ab115730; Abcam) and Alexa Fluor 488-labeled Goat Anti-Rabbit IgG (H + L) (1:500; A0423; Beyotime Biotechnology) antibodies. 4',6-diamidino-2-phenylindole (C1002; Beyotime Biotechnology) labeling was used to visualize cell nuclei. Then, visualization of the positively stained cells was performed using confocal laser scanning microscope (Leica Microsystems, Inc., Deerfield, IL, USA).

### Flow cytometry

Cells were seeded on 6-well plates. Cells were harvested 24 h later and washed with phosphate-buffered saline. Cells were then treated with FITC Mouse Anti-Human CD55 (561,900; BD Biosciences, Bedford, MA, USA) or Alexa Fluor^®^ 488 GLUT1 antibody (ab195359; Abcam). All readings were performed in triplicates and controlled using Alexa Fluor^®^ 488 Rabbit IgG (ab199091; Abcam) or FITC Mouse IgG2a, κ Isotype Control RUO (555,573; BD Biosciences) to address nonspecific labeling. Readings were compensated and analyzed using Becton–Dickinson FACScan Plus cytometer (Becton–Dickinson, San Jose, CA).

### Protein stability

To evaluate protein stability, cells transfected with the indicated plasmids were treated with 0.1 mg/ml cycloheximide (CHX; 239,765; Sigma-Aldrich, St. Louis, MO, USA) during indicated times and harvested. Protein quantity of GLUT1 was then determined by western blot analysis.

### In vivo tumor formation

In mice lung metastasis model, 5 × 10^6^ 253 J cells transduced with PIGT shRNA lentivirus were inoculated to each mouse. Otherwise, 5 × 10^6^ BIU-87 cells with transduced with PIGT overexpression lentivirus and/or GLUT1 shRNA lentivirus were inoculated to each mouse. Mice were sacrificed 6 weeks post injection, and lung was examined and collected for hematoxylin–eosin staining analysis. The number of metastatic nodules in lung was compared in each group. Mice were also collected for survival analysis.

### Analysis of m^6^A content

Total RNA was isolated by Trizol reagent (15,596,026; Thermo Fisher Scientific) and Poly(A)^+^ RNA was purified using GenElute™ mRNA Miniprep Kit (NMD70; Sigma-Aldrich). m6A content was estimated with m^6^A RNA Methylation Assay Kit (ab185912; Abcam) as previously described [25].

### Measurement of mRNA stability

Cells were treated by actinomycin D (SBR00013; Sigma-Aldrich) and collected for RNA extraction at 0, 3, and 6 h after treatment. mRNA levels were quantified by RT-qPCR as described above.

### RNA immunoprecipitation (RIP) assay

Magna RIP RNA-Binding Protein Immunoprecipitation Kit (17–700; Sigma-Aldrich) was used for the RIP assay following the manufacturer’s instructions. Cells were lysed and RNA–protein complexes were incubated with anti-m6A (ab208577; Abcam), anti-IGF2BP2 (ab128175; Abcam) or anti-IgG antibody (ab172730; Abcam) overnight at 4 °C and washed with RIP-wash buffer for 10 min at 4 °C and then RIP-lysis buffer for 5 min at 4 °C. The co-precipitated RNAs were purified using phenol:chloroform:isoamyl alcohol and subjected to RT-qPCR.

### Statistical analysis

All experiments were conducted at least three times independently. Data are presented as the mean ± SD and analyzed by GraphPad Prism 8.4.2 (GraphPad Software, San Diego, CA, USA). Comparisons were performed using student t-test or analysis of variance. Survival rate was calculated using the Kaplan–Meier method. *P* < 0.05 was represented statistically significance.

## Results

### PIGT is clinically relevant in patients with bladder cancer

**Fig. 1:**
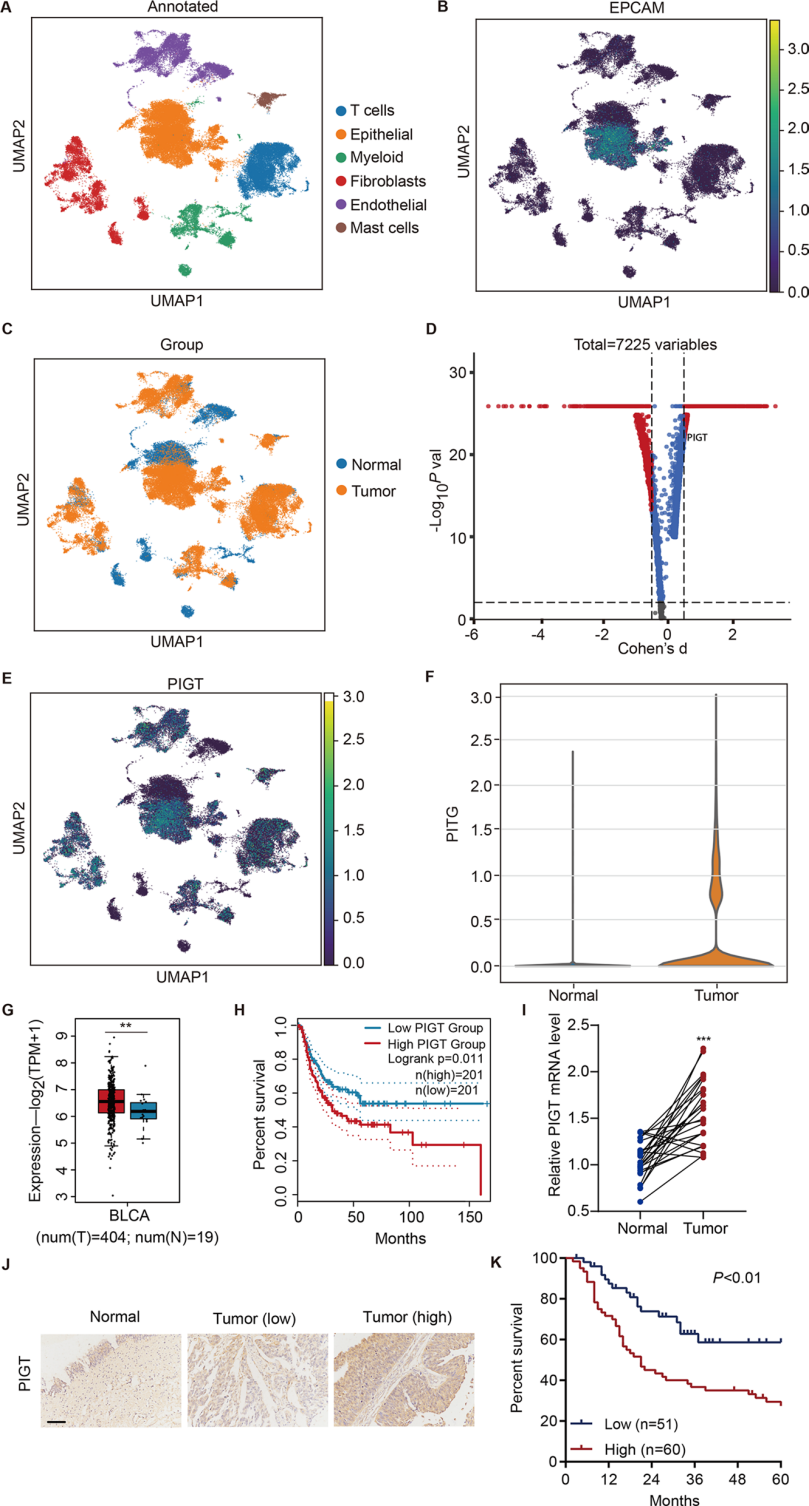
PIGT is clinically relevant in patients with bladder cancer. **A** UMAP plot of cell types in bladder cancer patients (PRJNA66201). **B** UMAP plot showing canonical marker genes for epithelial cells. **C** UMAP plot of tumor and normal tissue transcriptomes. **D** Gene expression of cancerous/normal cells by Volcano plot. **E** UMAP plot displayed the expression of PIGT. **F** Violin plot showed PIGT levels. **G** PIGT levels in cancer cells (T, red) and adjacent normal (N, blue) tissues in the TCGA database. **H** Survival analysis and comparison among people with high or low values of PIGT expression from TCGA database. **I** Results of PIGT expression in the hospital cohort (n = 25). **J** IHC staining of PIGT in bladder cancer microarrays. **K** Survival analysis and comparison among people with high or low PIGT in bladder cancer microarrays. ** *P* < 0.01, *** *P* < 0.001 vs normal (**N**)

To elaborate the clinical relevance of PIGT, single-cell RNA-seq data of bladder cancer was analyzed using UMAP for visualization. Cell type was labeled according to the expression of known markers (Fig. 1A), and the expression of canonical marker genes for epithelial cells was shown in Fig. 1B. UMAP was also used to plot the transcriptomes of tumor and normal tissue (Fig. 1C). Volcano plot showed the PIGT was one of the most significantly upregulated genes (Fig. 1D), which was also shown by UMAP (Fig. 1E). Data indicated that PIGT was remarkably up-regulated in bladder cancer (Fig. 1F). GEPIA indicated that expression of PIGT was increased in tumor compared with normal tissue (Fig. 1G) and high levels of PIGT were associated with shorter survival times of patients with bladder cancer in TCGA dataset (Fig. 1H). qRT-PCR data indicated that PIGT was sharply increased in bladder cancer tissues of hospital cohort (n = 25) (Fig. 1I). Then, the expression of PIGT in bladder cancer tissue microarrays was detected by IHC (Fig. 1J). Then, bladder cancer tissues were grouped into PIGT-high or PIGT-low group based on levels of PIGT. Patients with higher PIGT levels showed shorter survival times (Fig. 1K). Furthermore, the expression of PIGT was notably correlated with four of the clinicopathologic characteristics, pathological stage, grade, lymph node metastasis and vascular invasion, in the patients with bladder cancer (Additional file 1: Table S1). Moreover, GSEA data analysis showed the enrichment of KEGG_OXIDATIVE_PHOSPHORYLATION HALLMARK_GLYCOLYSIS and ALONSO_METASTASIS_UP pathways in subjects with high PIGT expression (Additional file 1: Fig S1A–C). These results demonstrate that PIGT is clinically relevant in bladder cancer.

### PIGT knockdown inhibits cell proliferation, oxidative phosphorylation and glycolysis in bladder cancer cells

**Fig. 2:**
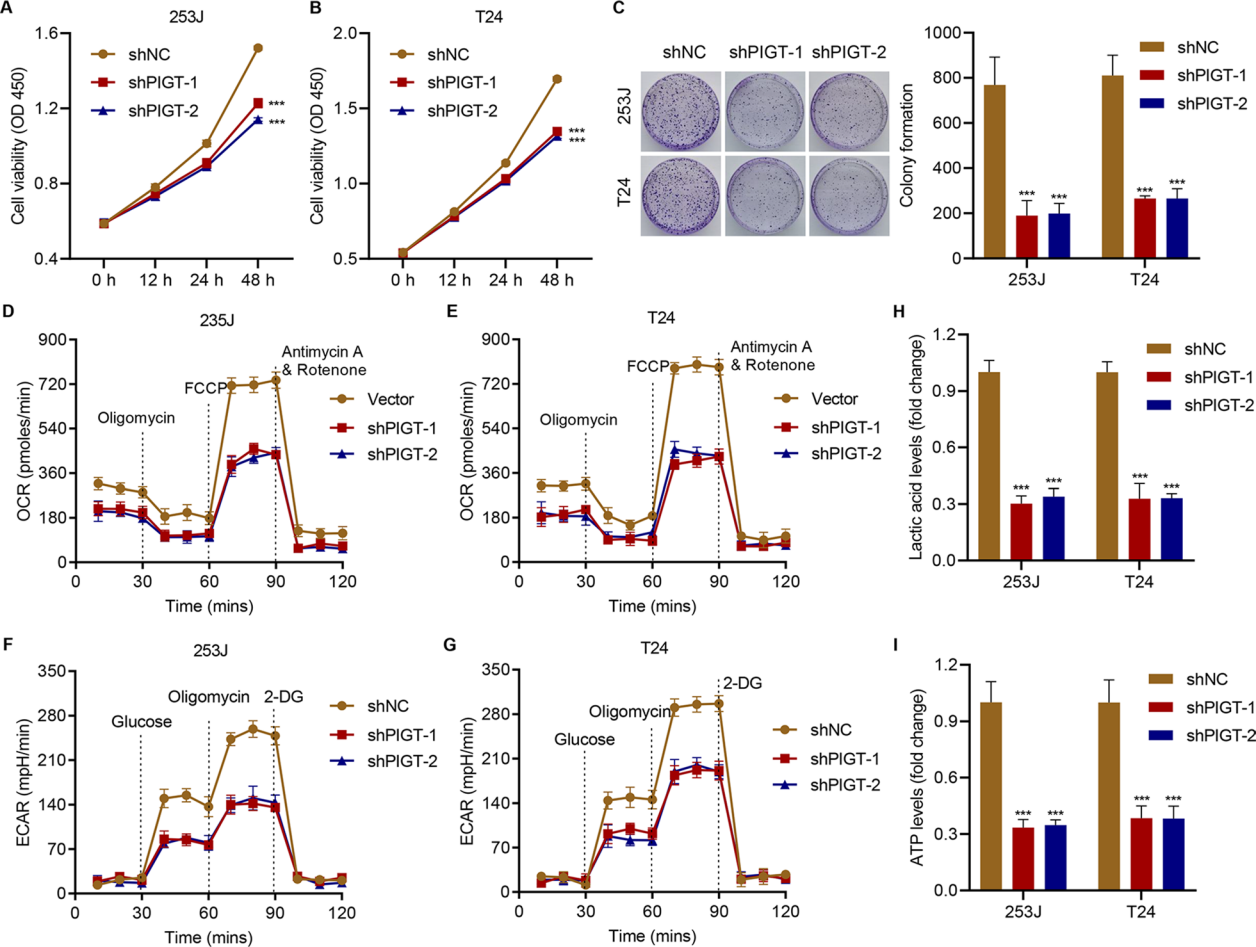
PIGT knockdown inhibits cell growth, oxidative phosphorylation and glycolysis in bladder cancer cells. 253 J and T24 cells were infected with lentivirus containing control shRNA (shNC) or PIGT shRNA (shRNA), and the **A**, **B** cell viability, **C** colony formation, **D**, **E** OCR, **F**, **G** ECAR, **H** lactic acid and **I** ATP levels were measured. ***P < 0.001 vs shNC

To investigate how PIGT is involved in bladder cancer, PIGT levels in a panel of bladder cancer cells was measured and data showed that two cell lines, 253 J and T24, have high levels of PIGT expression (Additional file 1: Fig S2A). Therefore, we silenced PIGT expression in the 253 J and T24 cells with the shRNA (Additional file 1: Fig S2B, C). To further examine the role of PIGT in regulating oxidative phosphorylation and glycolysis in bladder cancer, cell viability, colony formation, OCR, ECAR, ATP levels and lactic acid release were measured. Our data showed that silencing PIGT dramatically inhibited cell viability (Fig. 2A, B), colony formation (Fig. 2C), OCR (Fig. 2D, E), ECAR (Fig. 2F, G), and decreased lactic acid levels (Fig. 2H) and ATP levels (Fig. 2I) in 253 J and T24 cells. Together, the data indicate that PIGT knockdown inhibits cell proliferation, oxidative phosphorylation and glycolysis in bladder cancer cells.

### PIGT knockdown inhibits tumor metastasis

**Fig. 3:**
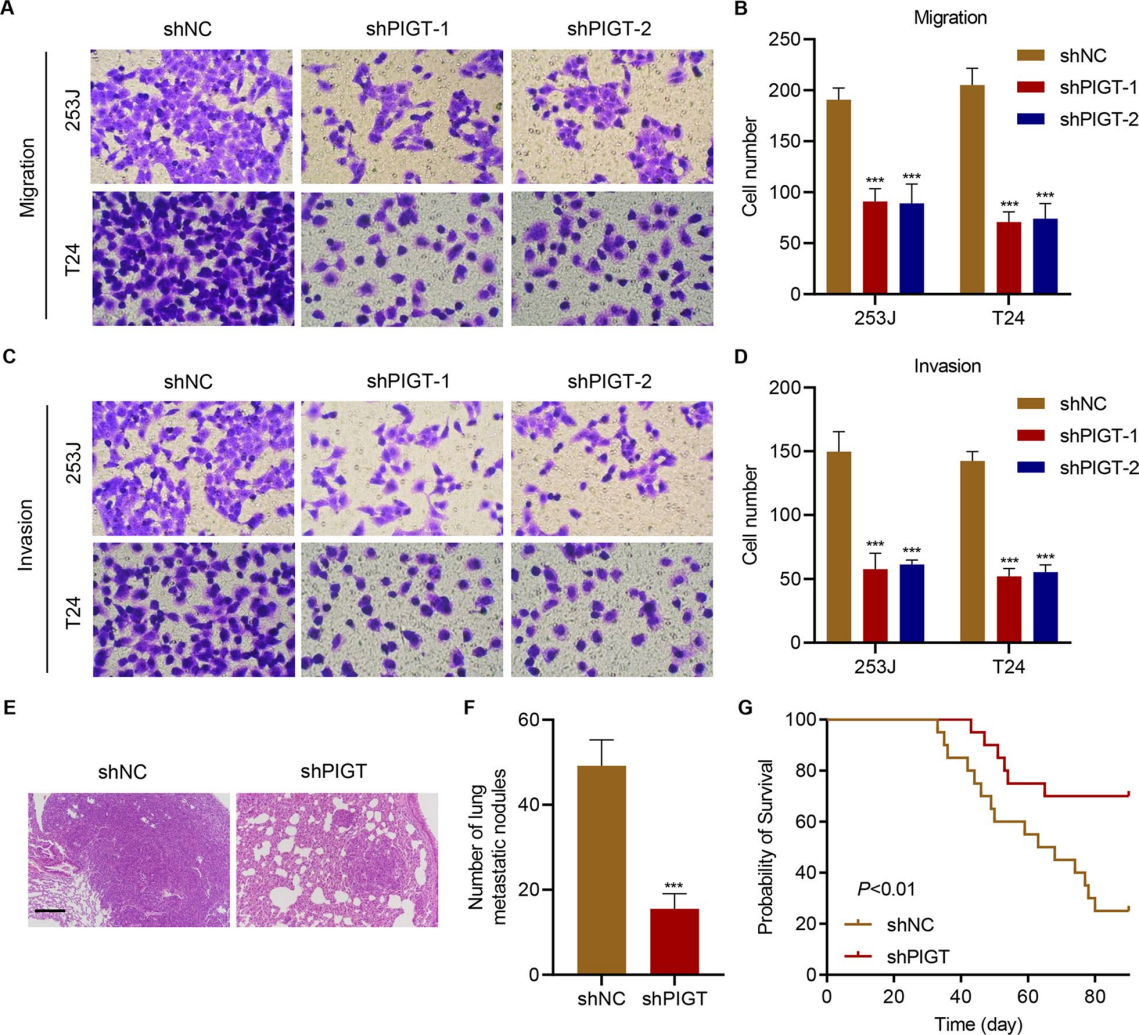
PIGT knockdown inhibits cell metastasis. 253 J and T24 cells were infected with lentivirus containing shNC or PIGT shRNA (shRNA), and the **A**, **B** migration and **C**, **D** invasion were detected. 253 J cells infected with shNC or PIGT shRNA lentivirus were injected to mice. **E** Histology of metastasized lungs of mice (scale bar, 200 μm). **F** Quantification of the number of metastatic nodules (6 mice in each group). **G** Survival rates of mice (20 mice in each group). ***P < 0.001 vs shNC

The effect of PIGT knockdown on cell migration and invasion in vitro was further investigated. Results showed that PIGT knockdown dramatically inhibited cell migration (Fig. 3A, B) and cell invasion (Fig. 3C, D) in 253 J and T24 cells. To further examine the role of PIGT in metastasis in vivo, lung metastasis model in bladder cancer-bearing mice was established. We found that PIGT knockdown also significantly inhibited tumor lung metastasis (Fig. 3E, F), and increased survival rates of tumor-bearing mice (Fig. 3G). These findings suggest that PIGT knockdown inhibits tumor metastasis.

### WTAP increases m6A modification of PIGT through IGF2BP2

**Fig. 4:**
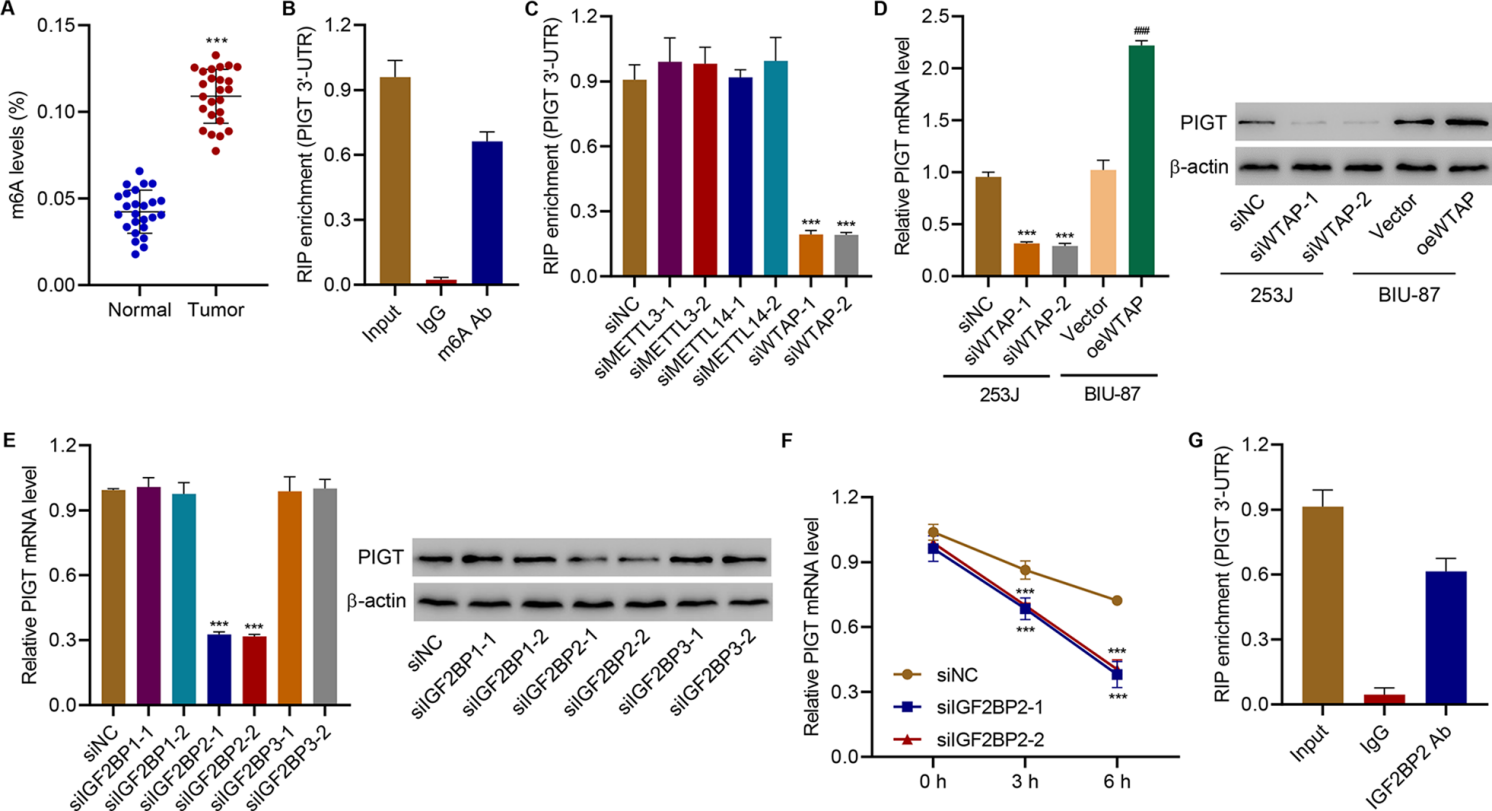
WTAP promotes the m6A modification of PIGT through IGF2BP2. **A** The global m^6^A level in bladder cancer tumor and normal tissues of the hospital cohort (n = 25). **B** m6A levels of PIGT 3′-UTR in 253 J cells. **C** m6A levels of PIGT 3′-UTR in 253 J cells transfected with METTL3, METTL14 or WTAP siRNA. **D** PIGT expression in 253 J-siWTAP and in BIU-87-vector or BIU-87-oeWTAP. **E** PIGT expression in 253 J cells transfected with IGF2BP1, IGF2BP2 or IGF2BP3 siRNA. **F** IGF2BP2 knockdown in 253 J cells decreased PIGT mRNA stability. **G** Interaction between IGF2BP2 and PIGT 3′UTR. ***P < 0.001 vs normal or siNC. ^###^*P* < 0.001 vs vector

m6A is the most prevalent post-transcriptional RNA modification [12], which is involved in the regulation of various physiological and pathological processes including bladder cancer [16, 17]. Given the increased mRNA and protein expression of PIGT in bladder cancer tissues and cell lines, we further investigated whether PIGT is regulated by m6A modification in bladder cancer. Results showed that m6A level was sharply increased in cancerous tissues (Fig. 4A). RIP followed by quantitative RT-qPCR assay results showed higher levels of m6A in PIGT 3′-UTR in 253 J cells (Fig. 4B). Enzymes referred to as m6A methyltransferases, such as the methyltransferase-like protein 3 (METTL3)-METTL14-Wilms’ tumor 1-associated protein (WTAP) complex, are responsible for adding m6A modifications [26]. Then, the PIGT 3′UTR enrichment in 253 J cells transfected with METTL3, METTL14 or WTAP siRNA was measured and results showed that silencing WTAP significant decreased levels of m6A in PIGT 3′UTR (Fig. 4C, Additional file 1: Fig S3). Data also supported that silencing WTAP significantly decreased PIGT expression, while overexpressing WTAP significantly increased PIGT expression (Fig. 4D, Additional file 1: Fig S3). To clarify which m6A reader played a role, insulin-like growth factor 2 mRNA-binding protein 1 (IGF2BP1), IGF2BP2, or IGF2BP3 was silenced in 253 J cells to study their effects on PIGT expression (Additional file 1: Fig S4). Results showed that silencing IGF2BP2 dramatically decreased levels of PIGT expression (Fig. 4E). IGF2BP2 knockdown in 253 J cells also decreased PIGT mRNA stability (Fig. 4F). RIP followed by quantitative RT-qPCR demonstrated that IGF2BP2 interacted with PIGT 3′UTR (Fig. 4G). Together, these findings demonstrated that WTAP increases m6A modification of PIGT through m6A reader, IGF2BP2.

### Post-translational modification of GLUT1 by PIGT

**Fig. 5:**
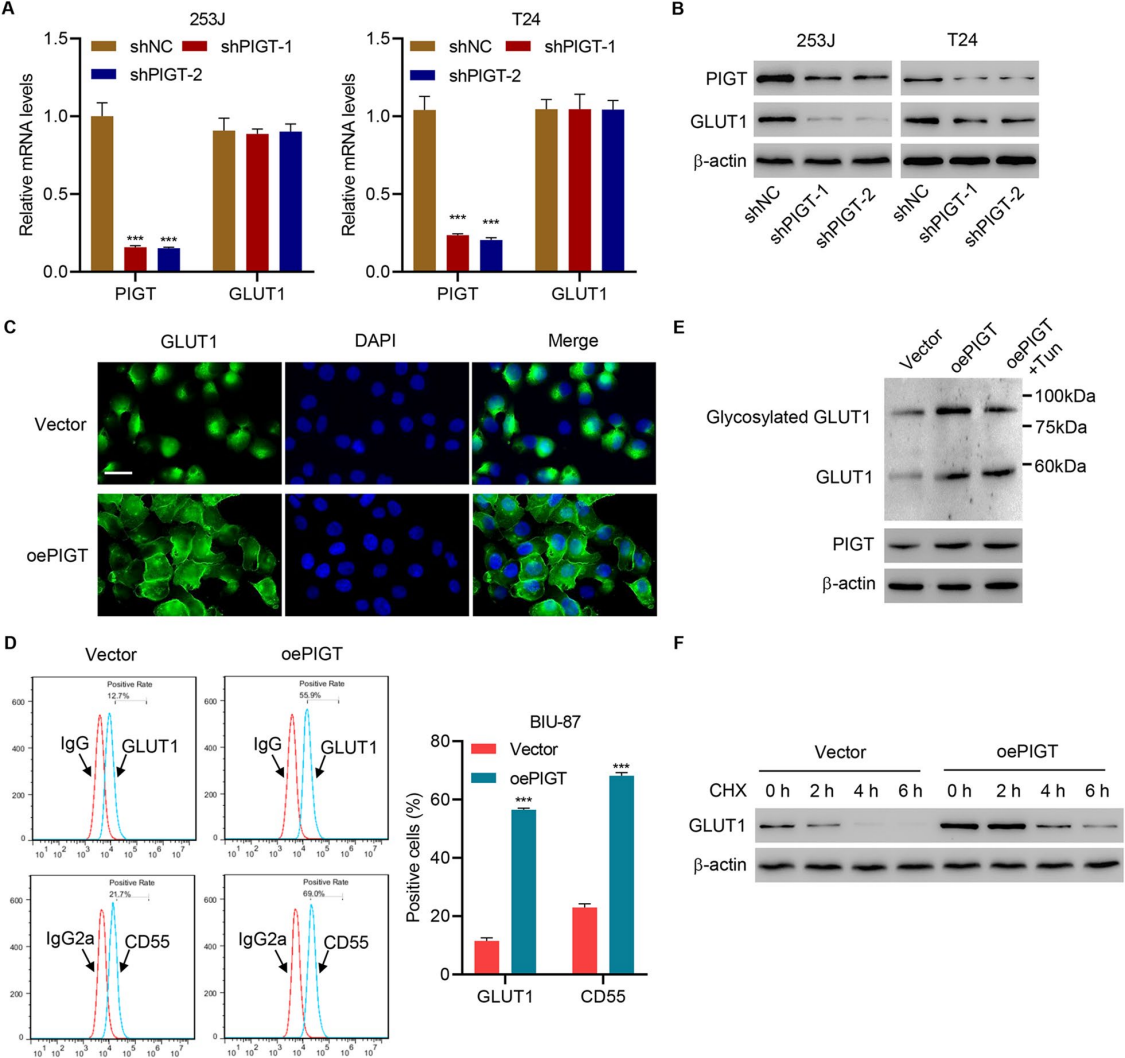
PIGT promotes GLUT1 glycosylation and membrane trafficking. **A**, **B** Expression of PIGT and GLUT1 in 253 J and T24 cells infected with shNC or PIGT shRNA. **C** Immunofluorescence for GLUT1 (green) in BIU-87 cells overexpressing PIGT (oePIGT) (scale bar, 50 μm). **D** FACS analysis CD55 membranous expression in BIU-87-oePIGT or BIU-87-vector. **E** Glycosylated and non-glycosylated GLUT1 in BIU-87-oePIGT or BIU-87-vector treated with tunicamycin (Tun). **F** GLUT1 expression levels in BIU-87 cells treated by cycloheximide. ***P < 0.001 vs shNC or vector

To examine the molecular mechanism by which PIGT regulates the development and progression of bladder cancer, the expression of key glucose transporter GLUT1 which has been reported to regulate tumorigenesis of bladder cancer was measured. Data showed that silencing PIGT did not affect GLUT1 at mRNA level (Fig. 5A), but decrease GLUT1 at protein level (Fig. 5B). Then, PIGT was successfully overexpressed in a PIGT-low-expressing cell line, BIU-87 (Additional file 1: Fig S2D). PIGT overexpression significantly promoted GLUT1 membrane trafficking (Fig. 5C). The expression of membranous GLUT1 is associated with post-translational modification [27]. At the endoplasmic reticulum, the crucial steps of cleaving the signal sequence and attaching the preassembled glycosylphosphatidylinositol (GPI) anchor are catalyzed by GPI transamidase (GPIT), a multisubunit membrane-bound enzyme [10]. PIGT is a subunit of GPIT, which facilitates the modification of proteins by GPI [28]. To address the role of the GPIT complex in GLUT1 expression and subcellular localization, we assessed the cell surface expression levels of GLUT1 and the canonical GPIT-dependent protein, decay-accelerating factor CD55, using flow cytometry. Overexpression of PIGT led to a marked increase in GPIT complex activity, as reflected by augmented CD55 expression, in parallel to a rise in the surface expression of GLUT1 (Fig. 5D).

The observation that PIGT expression affects GLUT1 trafficking to the membrane raises the question of whether GLUT1 is a GPI-anchored protein. We further evaluated the molecular structure of GLUT1 using the “big-PI” predictor algorithm (http://mendel.imp.ac.at/sat/gpi/gpi_server.html), in which sequence-analytical evidence reliably excludes GLUT1 as a possible substrate for GPI lipid anchoring.

Increasing evidences have shown that post-translational modifications such as glycosylation is implicated in transporter stability and membrane trafficking [29–31]. Therefore, we investigated whether PIGT influences the glycosylation of GLUT1. Immunoblotting results suggested that the increase of glycosylation of GLUT1 by PIGT overexpression was suppressed by administration of glycosylation inhibitor, tunicamycin (Tun) treatment (Fig. 5E). To assess GLUT1 protein stability, we performed immunoblot 2, 4, and 6 h after treating BIU-87 cells with a translational inhibitor cycloheximide (CHX). This analysis showed that PIGT overexpression significantly ameliorated CHX-induced decrease of GLUT1, suggesting an increased stability of GLUT1 protein (Fig. 5F). These findings demonstrate that post-translational modification of GLUT1 by PIGT influences GLUT1 expression.

### PIGT overexpression promotes cell proliferation, oxidative phosphorylation, and glycolysis by activating GLUT1

**Fig. 6:**
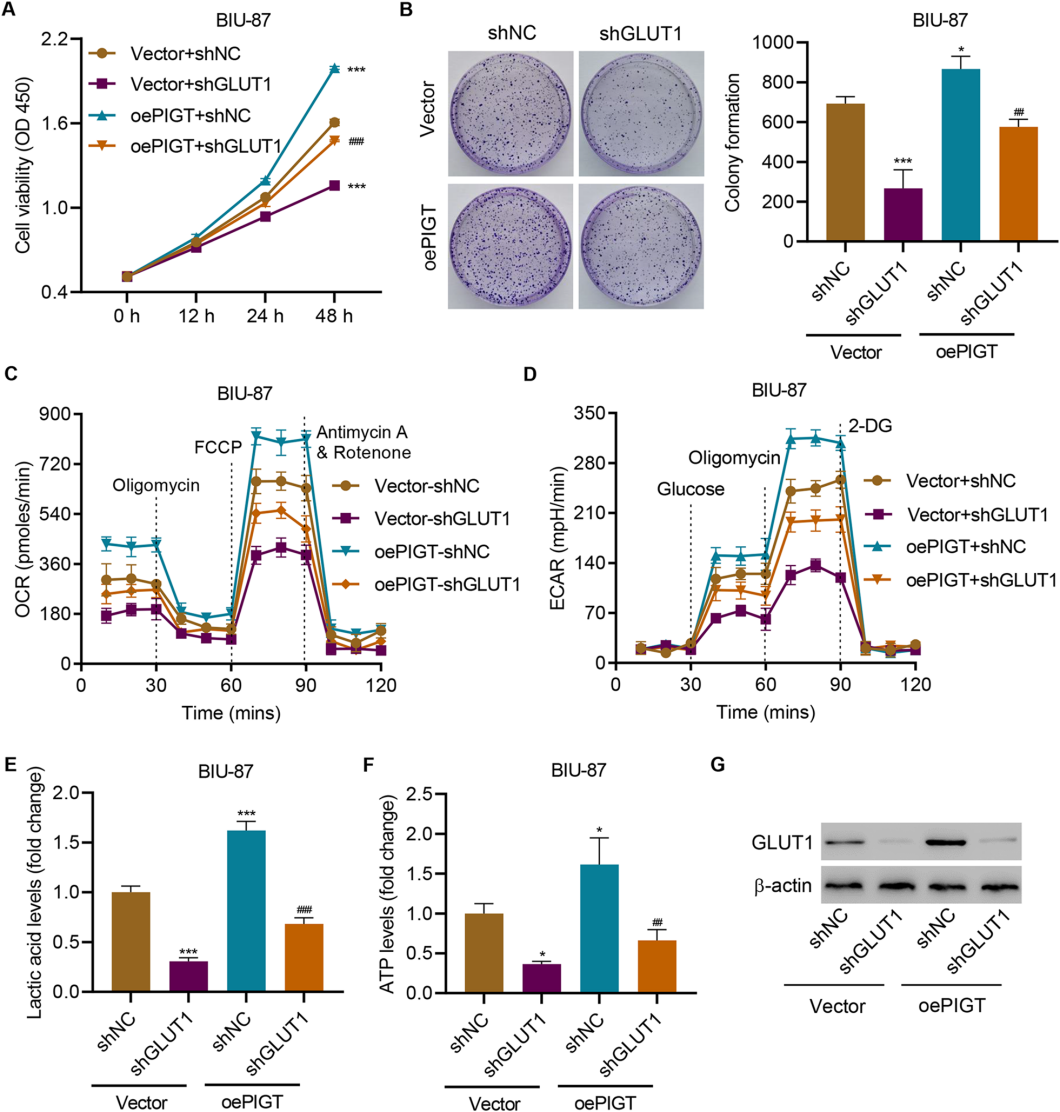
PIGT overexpression promotes cell growth, oxidative phosphorylation and glycolysis by activating GLUT1. BIU-87 cells were infected with control lentivirus (vector), PIGT overexpressing lentivirus (oePIGT) and/or GLUT1 shRNA lentivirus, and the (**A**) cell viability, (**B**) colony formation, (**C**) OCR, (**D**) ECAR, (**E**) lactic acid, (**F**) ATP levels and (**G**) GLUT1 expression were measured. * *P* < 0.05, *** *P* < 0.001 vs shNC + Vector. ^##^ *P* < 0.01, ^###^ *P* < 0.001 vs shNC + oePIGT

To further clarify the role of GLUT1 in regulating PIGT-induced cell proliferation, oxidative phosphorylation, and glycolysis, GLUT1 was silenced and PIGT was overexpressed in BIU-87 cells. PIGT overexpression significantly promoted cell viability, colony formation, OCR, and ECAR (Fig. 6A–D), which were all suppressed by silencing GLUT1. Furthermore, GLUT1 silencing also inhibited PIGT overexpression-induced increased levels of lactic acid (Fig. 6E) and ATP (Fig. 6F). Immunoblotting results showed that PIGT overexpression-increased GLUT1 at protein level (Fig. 6G). These findings demonstrate that GLUT1 is one of the mechanisms that how PIGT can promote the cell proliferation, oxidative phosphorylation, and glycolysis in bladder cancer.

### PIGT overexpression promotes tumor metastasis by activating GLUT1

**Fig. 7:**
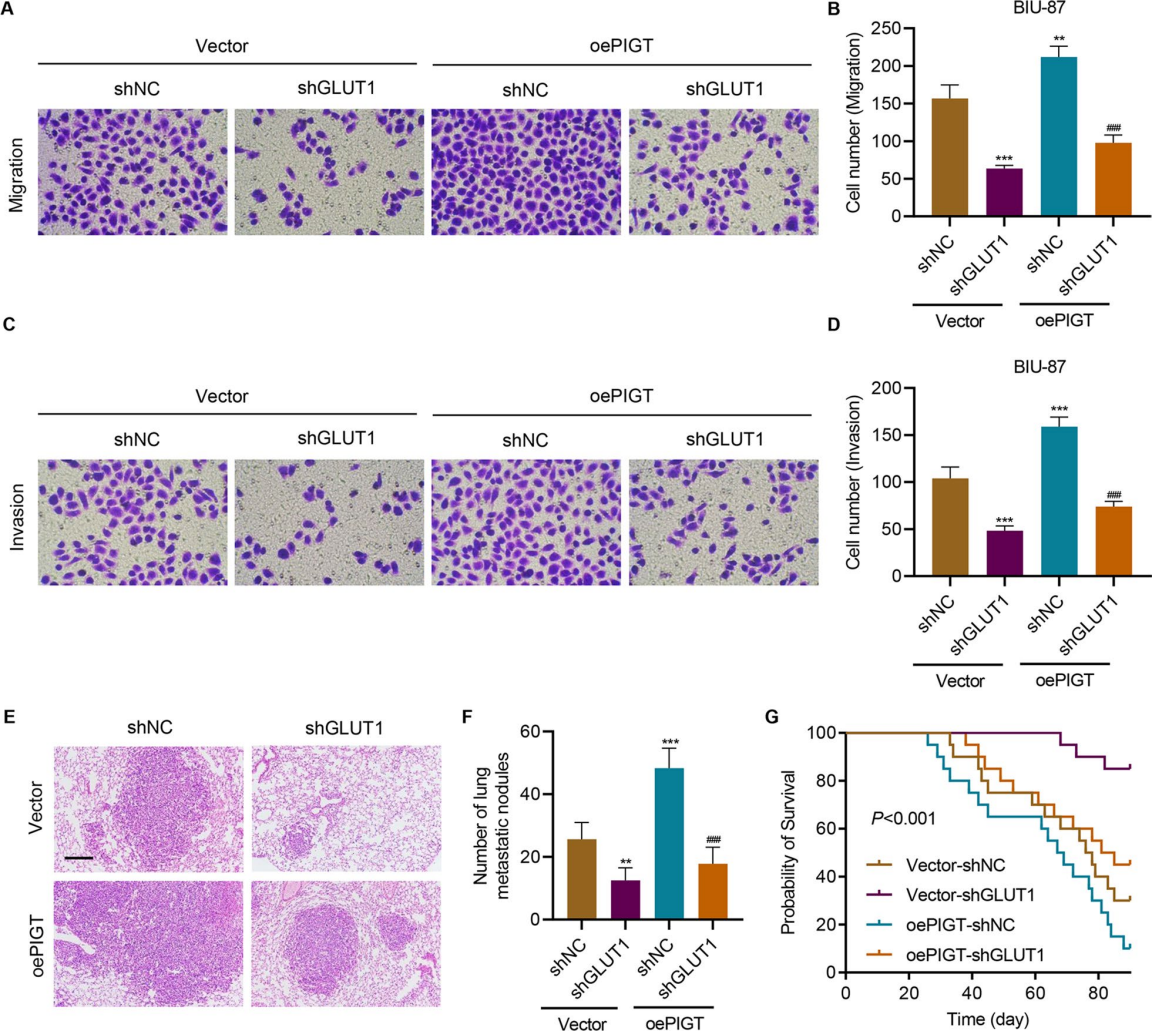
PIGT overexpression promotes cell metastasis by activating GLUT1. BIU-87 cells were transduced with control lentivirus (vector), PIGT overexpression lentivirus (oePIGT) and/or GLUT1 shRNA lentivirus, and the **A**, **B** cell migration and **C**, **D** invasion were measured. BIU-87-vector, BIU-87-oePIGT and/or GLUT1 shRNA lentivirus were given to mice. **E** Histology of metastasized lungs of mice (scale bar, 100 μm). **F** Quantification of the number of metastatic nodules (6 mice in each group). **G** Survival of mice (20 mice in each group). **P < 0.01, ***P < 0.001 vs shNC + Vector. ^###^*P* < 0.001 vs shNC + oePIGT

Further study showed that PIGT overexpression significantly enhanced cell migration (Fig. 7A, B) and invasion (Fig. 7C, D), which were all suppressed by silencing GLUT1. More importantly, in animal studies, GLUT1 silencing inhibited PIGT overexpression-induced tumor metastasis (Fig. 7E, F). GLUT1 silencing dramatically inhibited PIGT overexpression-induced decreased survival rates of tumor-bearing mice (Fig. 7G). These results indicate that PIGT overexpression promotes tumor metastasis by activating GLUT1.

### Correlation analysis of PIGT, WTAP, and GLUT1 in bladder cancer

**Fig. 8:**
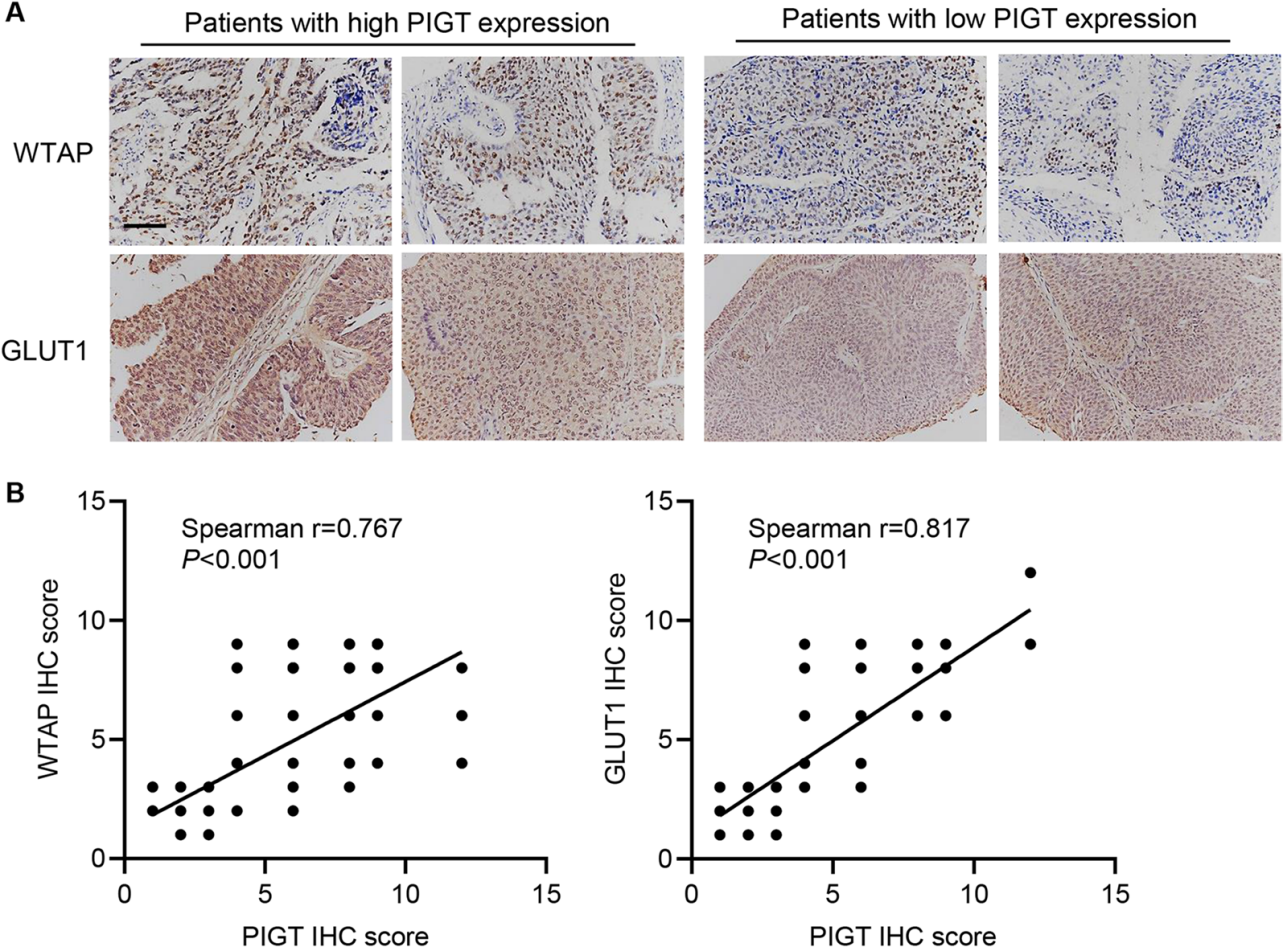
Correlation analysis of PIGT, WTAP, and GLUT1 in bladder cancer. **A** Representative IHC images of WTAP and GLUT1 in bladder cancer tissue microarrays (scale bar, 100 μm). **B** Correlation analysis of PIGT, WTAP, and GLUT1 expression in bladder cancer tissue microarrays

To determine the relevance of the regulation of PIGT, WTAP and GLUT1 in patients, we performed IHC staining of PIGT, WTAP, and GLUT1 on the bladder cancer tissue microarrays and tumor tissues were separated into PIGT-high/low-group (Fig. 8A). Moreover, correlation analysis demonstrated that PIGT expression was positively correlated with WTAP and GLUT1 expression in bladder cancer tissues (Fig. 8B).

## Discussion

We have demonstrated that silencing PIGT suppresses cell growth, oxidative phosphorylation, and glycolysis in bladder cancer cells, whereas overexpressing PIGT promotes cell proliferation, oxidative phosphorylation, and glycolysis via activating GLUT1. Further studies showed that silencing PIGT inhibits metastasis, while overexpressing PIGT promotes metastasis in vivo*.* Mechanism study indicated that WTAP increases m6A modification of PIGT through IGF2BP2 to enhance the stability of PIGT. For the first time, our study indicated that m6A RNA methylation-mediated PIGT promotes cell proliferation, oxidative phosphorylation, and glycolysis by modulating GLUT1 glycosylation and membrane trafficking.

PIGT controls the adding of GPI anchors to proteins [28]. Wu et al. have demonstrated that overexpressing PIGT induced tumorigenesis and promoted the invasiveness of breast cancer [8]. PIGT’s counterpart, PIGU, has been shown to be increased in bladder cancer, and more importantly, study showed that overexpressing PIGU malignantly transformed NIH3T3 cells [32]. Cao et al. pointed out that considering PIGU expression could improve the prognostic stratification of hepatocellular carcinoma patients [11]. Compare to PIGU, the role of PIGT in cancer, especially in bladder cancer, is poorly understood. In the current study, we revealed a new role of PIGT in bladder cancer, showing that PIGT regulates cell proliferation, oxidative phosphorylation, and glycolysis in bladder cancer cells.

Metabolic abnormality is a hallmark of cancer and cancer cells use aerobic glycolysis to metabolize glucose [33]. Under hypoxia, cancer cells often increase levels of GLUTs, especially GLUT1 and GLUT3 [34]. It has been shown that bladder cancer cells rely on aerobic glycolysis as the main energy source [35]. Li et al. have reported that miR-218 increased the sensitivity of bladder cancer to cisplatin by targeting Glut1 [36]. Expression of GLUT1 was associated with increasing grade of bladder cancer [37]. It also has been reported that induction of GLUT1 increased the glucose uptake rate [38]. Here, we demonstrated that GLUT1 silencing significantly suppressed proliferation, oxidative phosphorylation, and glycolysis.

Post-translational modification is an important regulator of protein function and an important step of signal transduction [39]. In cancer cells, post-translational modification of effector proteins can result in abnormally fast cell proliferation [39]. It has been shown that GLUT1 is increased in some cancer and is regulated by post-translational modification [40]. As one important form of post-translational modification, glycosylation has been shown to influence cancer progression, and abnormal glycosylation regulates cell grwoth, and invasion [41]. However, the role of glycosylation of GLUT1 in cancer especially bladder cancer remains to be elucidated. Here, we demonstrated that PIGT increased GLUT1 glycosylation and membrane trafficking, leading to enhanced cell proliferation, oxidative phosphorylation, glycolysis, and metastasis. Together, these results revealed a new role of GLUT1 glycosylation, suggesting that increasing GLUT1 glycosylation and membrane trafficking can promote bladder cancer progression which might provide a scientific basis for new drug development.

m6A has been associated to cellular differentiation, cancer progression, etc. [14]. m6A is formed by m6A methyltransferases (WTAP etc., termed “writers”), removed by demethylases, and read by readers (IGF2BP1/2/3 etc.) [42]. WTAP recruits m^6^A methyltransferase to targets [43]. WTAP has been shown to facilitate progression of liver cancer via m6A-HuR-dependent silencing of ETS1 [44]. WTAP also m6A-dependently promoted osteosarcoma tumorigenesis [45]. In bladder cancer, WTAP increased the activity of m6A methyltransferase and increased cisplatin resistance [46]. Chen et al. have reported that WTAP was dramatically up-regulated in bladder cancer patients [47]. Here we showed for the first time that WTAP was involved in the m^6^A of modification of PIGT mRNA, leading to upregulation of PIGT expression. Overexpression of WTAP resulted in up-regulation of PIGT probably through enhancement of PIGT mRNA stability. We also found that WTAP increases m6A modification of PIGT through IGF2BP2. The results indicate a novel role of WTAP in bladder cancer, demonstrating that WTAP increases the m6A modification of PIGT through IGF2BP2 to contribute to bladder cancer progression. Certain limitations exist. For instance, experiments were carried out in animals and cells. Experiments using PDX models or clinical specimen can provide more persuasive results. Nevertheless, we show a novel mechanism underlying proliferation and metastasis of bladder cancer and may provide novel directions for drug development.

## Conclusions

This study demonstrated a novel function of PIGT, showing that PIGT enhances cell growth, glycolysis, and metastasis in bladder cancer by modulating GLUT1 glycosylation and membrane trafficking and that WTAP increases m6A modification of PIGT through m6A reader, IGF2BP2.

### Supplementary Information

**Additional file 1:** **Table S1.** Clinicopathological features of 111 bladder cancer patients and the expression of PIGT. **Table S2.** The sequences of shRNAs/siRNAs used in the study. **Table S3.** Primer sequences used in the study. **Figure S1.** GSEA data analysis. Results showed the enrichment of **A** KEGG_OXIDATIVE_PHOSPHORYLATION, **B** HALLMARK_GLYCOLYSIS and **C** ALONSO_METASTASIS_UP peaks in subjects with high PIGT expression compared with low PIGT expression. **Figure S2.** PIGT expression in bladder cancer cell lines. **A** Expression of PIGT in 253 J, 5637, BIU-87, T24, SCABER, and SV-HUC-1. **B**, **C** Expression of PIGT in 253 J and T24 cells transduced with PIGT shRNA or control shRNA (shNC). **D** Expression of PIGT in BIU-87 cells transduced with PIGT overexpression lentivirus or blank vector. ***P < 0.001 vs SV-HUC-1, shNC1 or vector. **Figure S3. **METTL3, METTL14 and WTAP expression in bladder cancer cell lines. Levels of **A** METTL3, **B** METTL14 and **C** WTAP in 253 J cells transfected with control siRNA (siNC) or METTL3, METTL14, or WTAP siRNA. **D** Expression of WTAP in BIU-87 cells transduced with WTAP overexpression lentivirus or blank vector. ***P < 0.001 siNC or vector. **Figure S4.** IGF2BP1, IGF2BP2 and IGF2BP3 expression in bladder cancer cell lines. Levels of IGF2BP1-3 in 253 J cells transfected with control siRNA (siNC) or IGF2BP1, IGF2BP2, or IGF2BP3 siRNA. ***P < 0.001 vs siNC.

## Data Availability

All data presented in this study are included within the paper and its Supplementary files.

## Abbreviations

GPI: Glycosylphosphatidylinositol
GPIT: Glycosylphosphatidylinositol transamidase
PIGT: Phosphatidylinositol glycan anchor biosynthesis class T
m6A: N6-methyladenosine
GLUT1: Glucose transporter 1
UMAP: Uniform manifold approximation and projection
TCGA: The Cancer Genome Atlas
GEPIA: Gene Expression Profiling Interactive Analysis
GSEA: Gene set enrichment analysis
TPM: Transcript per million
IHC: Immunohistochemistry
CCK-8: Cell Counting Kit-8
OCR: Oxygen consumption rate
ECAR: Extracellular acidification rate
RT-qPCR: Quantitative RT-PCR
CHX: Cycloheximide
RIP: RNA immunoprecipitation
METTL3: Methyltransferase-like protein 3
WTAP: Wilms’ tumor 1-associated protein
Tun: Tunicamycin
IGF2BP: Insulin-like growth factor 2 mRNA-binding protein
