# Supplementary material for: PIGT promotes cell growth, glycolysis, and metastasis in bladder cancer by modulating GLUT1 glycosylation and membrane trafficking
Source: J Transl Med. 2024 Jan 2;22:5. doi: 10.1186/s12967-023-04805-0 (PMC10763284; doi:10.1186/s12967-023-04805-0)
Supplement: Supplementary file 1 — Additional file 1: Table S1. Clinicopathological features of 111 bladder cancer patients and the expression of PIGT. Table S2. The sequences of shRNAs/siRNAs used in the study. Table S3. Primer sequences used in the study. Figure S1. GSEA data analysis. Results showed the enrichment of A KEGG_OXIDATIVE_PHOSPHORYLATION, B HALLMARK_GLYCOLYSIS and C ALONSO_METASTASIS_UP peaks in subjects with high PIGT expression compared with low PIGT expression. Figure S2. PIGT expression in bladder cancer cell lines. A Expression of PIGT in 253 J, 5637, BIU-87, T24, SCABER, and SV-HUC-1. B, C Expression of PIGT in 253 J and T24 cells transduced with PIGT shRNA or control shRNA (shNC). D Expression of PIGT in BIU-87 cells transduced with PIGT overexpression lentivirus or blank vector. ***P < 0.001 vs SV-HUC-1, shNC1 or vector. Figure S3. METTL3, METTL14 and WTAP expression in bladder cancer cell lines. Levels of A METTL3, B METTL14 and C WTAP in 253 J cells transfected with control siRNA (siNC) or METTL3, METTL14, or WTAP siRNA. D Expression of WTAP in BIU-87 cells transduced with WTAP overexpression lentivirus or blank vector. ***P < 0.001 siNC or vector. Figure S4. IGF2BP1, IGF2BP2 and IGF2BP3 expression in bladder cancer cell lines. Levels of IGF2BP1-3 in 253 J cells transfected with control siRNA (siNC) or IGF2BP1, IGF2BP2, or IGF2BP3 siRNA. ***P < 0.001 vs siNC. [file 12967_2023_4805_MOESM1_ESM.docx]

**Table S1.** **Clinicopathological features of 111 bladder cancer patients and the expression of PIGT**

| Parameters | Group | Cases | PIGT low | PIGT high | *P*-value |
| --- | --- | --- | --- | --- | --- |
| Age at surgery | <55 | 53 | 25 | 28 | 0.8047 |
|  | ≥55 | 58 | 26 | 32 |  |
| Gender | Male | 67 | 33 | 34 | 0.3882 |
|  | female | 44 | 18 | 26 |  |
| Pathological stage | pTa-T1 | 42 | 26 | 16 | 0.0085 |
|  | pT2-T4 | 69 | 25 | 44 |  |
| Grade | Low | 48 | 28 | 20 | 0.0223 |
|  | High | 63 | 23 | 40 |  |
| Lymph node metastasis | Absent | 62 | 21 | 41 | 0.0041 |
|  | Present | 49 | 30 | 19 |  |
| Vascular invasion | Absent | 72 | 27 | 45 | 0.0153 |
|  | Present | 39 | 24 | 15 |  |

*P* < 0.05 represents statistical significance (Chi-square test).

**Table S2. The sequences of shRNAs/siRNAs used in the study**

| shRNA/siRNA | Sequence (5’-3’) |
| --- | --- |
| shNC | GGTTAAGGCTTCATACACA |
| shPIGT-1 | CCACTACTTTCTGCGCTAT |
| shPIGT-2 | GCCGAGTCTATGTGGACAT |
| shGLUT1-1 | GCTCATGGGCTTCTCGAAA |
| shGLUT1-2 | GCCTGTGTATGCCACCATT |
| siNC | CAGUACUUUUGUGUAGUACAA |
| siMETTL3-1 | GCUGCACUUCAGACGAAUUTT |
| siMETTL3-2 | GGAUACCUGCAAGUAUGUUTT |
| siMETTL14-1 | GCAUUGGUGCCGUGUUAAATT |
| siMETTL14-2 | GCUGACAGAUUUGAAGAAUTT |
| siWTAP-1 | GCAAGUACACAGAUCUUAATT |
| siWTAP-2 | GCGAAGUGUCGAAUGCUUATT |
| siIGF2BP1-1 | GGACUUGGAGAAAGUGUUUTT |
| siIGF2BP1-2 | GGCUCAGUAUGGUACAGUATT |
| siIGF2BP2-1 | CCCAGUUUGUUGGUGCCAUTT |
| siIGF2BP2-2 | GCGAAAGGAUGGUCAUCAUTT |
| siIGF2BP3-1 | CCUUGAAAGUAGCCUAUAUTT |
| siIGF2BP3-2 | GCUGCUGAGAAGUCGAUUATT |

**Table S3. Primer sequences used in the study**

| Gene | Forward/Reverse | Sequence (5’-3’) |
| --- | --- | --- |
| PIGT | Forward | CAAGGGCAAGGAGAACAAAC |
|  | Reverse | CGCTCAAACTGGATGGAAAC |
| GLUT1 | Forward | TGCAGGAGATGAAGGAAG |
|  | Reverse | CAATGGTGGCATACACAG |
| METTL3 | Forward | CCTTTGCCAGTTCGTTAGTC |
|  | Reverse | TCCTCCTTGGTTCCATAGTC |
| METTL14 | Forward | CTGGGAATGAAGTCAGGATAG |
|  | Reverse | CCAGGGTATGGAACGTAATAG |
| WTAP | Forward | AAAGCAGTGAGTGGGAAAG |
|  | Reverse | AGCGGCAGAAGTATTGAAG |
| IGF2BP1 | Forward | ATGGAGTGTTTAGCCCTTGTG |
|  | Reverse | TGTTGCGGTTGTCTTGTTG |
| IGF2BP2 | Forward | CGGGAGCAAACCAAAGACC |
|  | Reverse | GCAAACCTGGCTGACCTTC |
| IGF2BP3 | Forward | GCACTTCCCTTTGTTGTAGTC |
|  | Reverse | AGCACTTCCCTTAGGTTACTC |
| β-actin | Forward | GATGACCCAGATCATGTTTGAG |
|  | Reverse | TAATGTCACGCACGATTTCC |
| PIGT 3’-UTR-m6A | Forward | CAGTACAGGAGCCACGAGC |
|  | Reverse | CAATACAGCAGCCACCGAC |


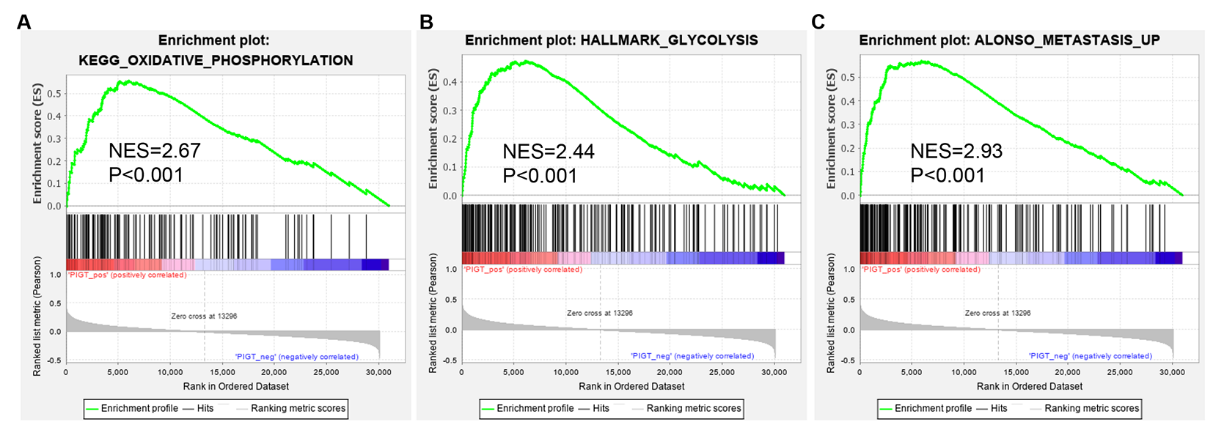


**Figure S1.** **GSEA data analysis.** Results showed the enrichment of (A) KEGG_OXIDATIVE_PHOSPHORYLATION, (B) HALLMARK_GLYCOLYSIS and (C) ALONSO_METASTASIS_UP peaks in subjects with high PIGT expression compared with low PIGT expression.


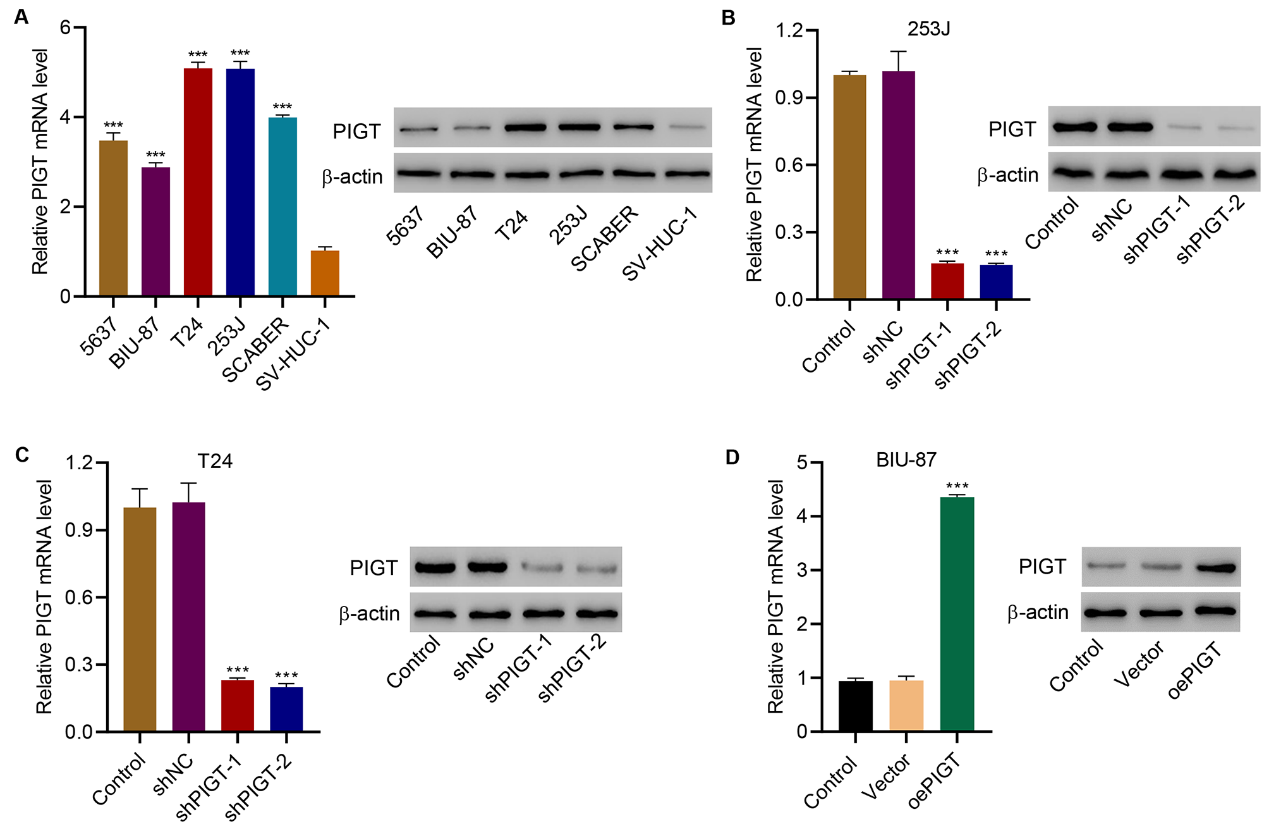


**Figure S2. PIGT expression in bladder cancer cell lines.** (A) Expression of PIGT in 253J, 5637, BIU-87, T24, SCABER, and SV-HUC-1. (B, C) Expression of PIGT in 253J and T24 cells transduced with PIGT shRNA or control shRNA (shNC). (D) Expression of PIGT in BIU-87 cells transduced with PIGT overexpression lentivirus or blank vector. ****P*<0.001 vs SV-HUC-1, shNC1 or vector.


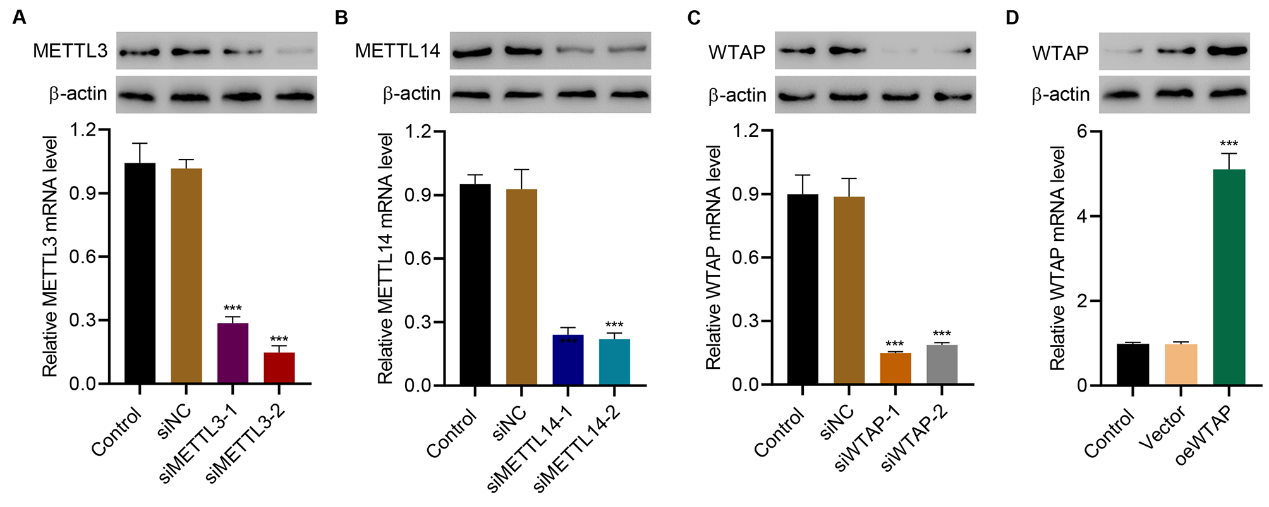


**Figure S3. METTL3, METTL14 and WTAP expression in bladder cancer cell lines.** Levels of (A) METTL3, (B) METTL14 and (C) WTAP in 253J cells transfected with control siRNA (siNC) or METTL3, METTL14, or WTAP siRNA. (D) Expression of WTAP in BIU-87 cells transduced with WTAP overexpression lentivirus or blank vector. *** *P* < 0.001 siNC or vector.


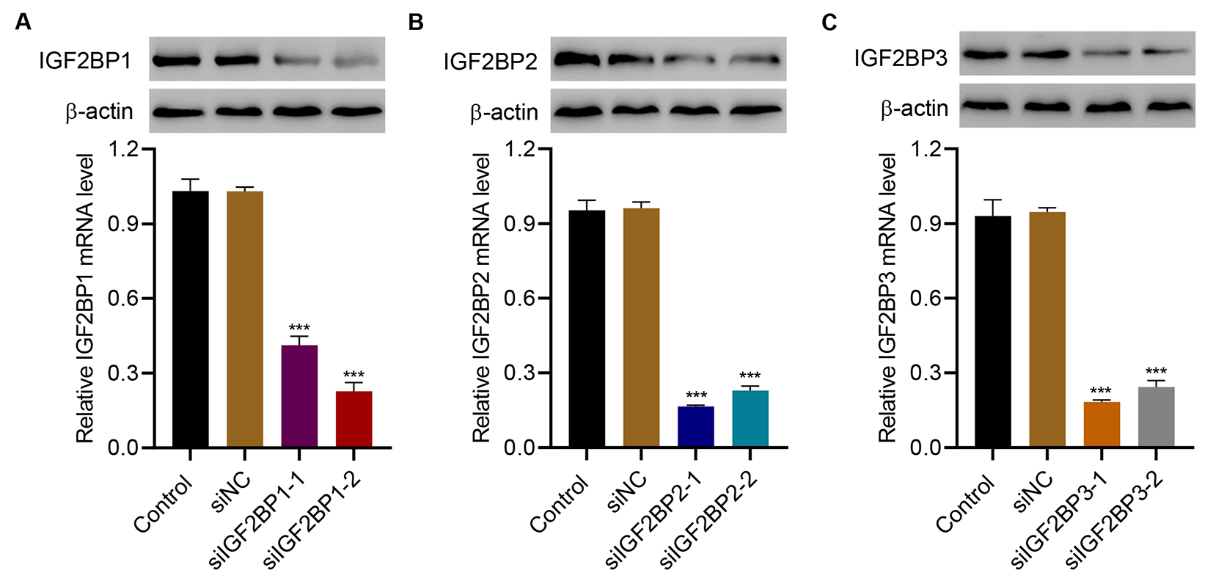


**Figure S4. IGF2BP1, IGF2BP2 and IGF2BP3 expression in bladder cancer cell lines.** Levels of IGF2BP1-3 in 253J cells transfected with control siRNA (siNC) or IGF2BP1, IGF2BP2, or IGF2BP3 siRNA. ****P*<0.001 vs siNC.
